# Supplementary material for: WordCommentsAnalyzer: A windows software tool for qualitative research
Source: F1000Res. 2018 Sep 4;7:536. Originally published 2018 May 3. [Version 2] doi: 10.12688/f1000research.14819.2 (PMC6137411; doi:10.12688/f1000research.14819.2)
Supplement: Supplementary file 3 [file f1000research-7-17614-s0002.tgz › b2a79496-4ada-47c4-9682-a99ce867836d.zip!/Eight Iranian Health Journals/The Journal of Toloo-e-Behdasht_34.docx]

© The Journal of Toloo-e-Behdasht. This work is licensed under CC BY 4.0 (https://creativecommons.org/licenses/by/4.0/).

Volume & Issue: دوره 11 - شماره 2

Title: ررسی DMFT دندان های مولر اول دائمی در دانش‌آموزان مدارس ابتدایی شهر یزد

http://tbj.ssu.ac.ir/article-1-177-fa.html

سابقه و اهداف: مولر اول دائمی بزرگترین دندان دائمی بوده و مهم ترین نقش را در جویدن، صحیح قرار گرفتن سایر دندانهای دائمی، برقراری اکلوژن، حفظ ارتفاع عمودی فکین و زیبایی دارد از طرفی به دلیل داشتن فرم آناتومیک خاص، رویش زود هنگام، عدم اطلاع مادر از رویش آن و اشتباه گرفتن با دندان شیری، بسیار مستعد پوسیدگی است. پوسیدگی در این دندان به عنوان شاخصی برای تشخیص پوسیدگی در دندان‌ها است. این مطالعه به منظور بررسی شیوع DMFT در دانش آموزان مدارس ابتدایی شهر یزد انجام گردیده است.   روش بررسی: در این مطالعه، دانش آموزان مجموعا 6 مدرسه دولتی و غیر انتفاعی به طور تصادفی انتخاب شدند و دندانهای مولر اول 15 دانش آموز از هر پایه مورد معاینه قرار گرفت و TDMF دندان های مولر اول دائمی آنها ثبت شده، سپس از طریق نرم افزار SPSS و تست‌های ANOVA و T-test مورد آنالیز قرار گرفت.   یافته‌ها: کمترین DMFT در گروه سنی 7 سال (92/0 ± 36/0) و بیشترین در گروه سنی 11سال (26/1 ± 17/1) بود. میان سن و DMFT رابطه معنی داری وجود داشت (0001/0 < P ).بین شاخص M,D,F بیشترین میانگین متعلق به F یا دندانهای پرشده (72/0 ± 13/1) و کمترین مقدار متعلق به M یا دندانهای کشیده شده (25/0 ± 04/0) بود.دخترها DMFT بالاتری (33/1 ± 15/1) از پسران (88/0 ± 51/0) داشتند (05/0 P< ). DMFT در دانش آموزان با وضعیت اقتصادی پایین، به طور معناداری بالاتر از دانش آموزان با وضعیت اقتصادی بهتر بود.   نتیجه گیری: افزایش DMFT طی 4-3 سال پس از رویش در دندانهای مولر اول دائمی لزوم توجه بیشتر را به امر آموزش، پیشگیری و درمان زود هنگام نشان می دهد.

Title: بررسی و مقایسه اثر آموزش کلامی– تصویری و آموزشهای روتین بر وابستگی به نیکوتین در بیماران سیگاری پس از عمل بای پاس عروق کرونر

http://tbj.ssu.ac.ir/article-1-178-fa.html

سابقه واهداف: مصرف سیگار سالیانه بیش از 5 میلیون مرگ را در جهان در پی دارد و سهم آن برای کشورهای توسعه یافته و در حال توسعه به ترتیب 30 درصد و 70 درصد می باشد. مهمترین و قابل پیشگیری ترین عامل برای مرگ و سکته های قلبی، ترک سیگار است.با وجود خطر درک شده ، هنوز هم 40 درصد از سیگاریهایی که یک حمله قلبی داشته اند، در روزهای بعد از ترک بیمارستان، سیگار کشیدن را از سرگرفته اند که این امر نشان دهنده کافی نبودن آموزش های صورت گرفته می باشد .هدف ازاین مطالعه بررسی و مقایسه اثر آموزش کلامی– تصویری و آموزشهای روتین بر وابستگی به نیکوتین در بیماران مرد پس از عمل بای پاس عروق کرونر می باشد.   روش بررسی: این مطالعه تحلیلی به روش کارآزمایی بالینی برروی100 نفر ازبیماران مرد کاندید عمل جراحی قلب بستری در بیمارستان افشار یزد انجام گردید. بیماران بصورت تصادفی به دوگروه مداخله و شاهد تقسیم شدند که اطلاعات آنها در دو مرحله قبل وبعد از عمل جراحی واز طریق دوپرسشنامه جمع آوری گردید.پرسشنامه مرحله اول شامل اطلاعات دموگرا فیک، تاریخ عمل،آدرس، تلفن، تست استاندارد وا بستگی به نیکوتین ( فاگروشتروم ) و تست استاندارد خود کارا مدی (پروچسکا – دی کلمنت) بود. پس از تکمیل پرسشنامه مرحله اول،گروه مداخله تحت آموزش روتین وآموزش کلامی ترک سیگار به همراه سه تصویر رنگی واضح از گرفتگی عروق به مدت 5 دقیقه قرار گرفتند وگروه شاهد تنها تحت آموزش روتین قرار گرفتند. سه ماه بعداز عمل ضمن تماس با بیماران ، پرسشنامه مرحله دوم تکمیل گردید .این پرسشنامه نیز شامل سوالاتی در رابطه با ترک یا عدم ترک سیگار، میل به سیگار ، زمان شروع سیگار بعد از عمل ،پرسشنامه فاگروشتروم و خودکارامدی بود .سپس داده های دومرحله توسط نرم افزار spss وتست های موردنیاز تجزیه وتحلیل گردید.   یافته ها : دوگروه مداخله وشاهد از لحاظ متغیرها ی دموگرافی یکسان بودند. میانگین سنی گروه مداخله وشاهد به ترتیب 9/8 ± 55/53 و 4/8 ± 6/55 سال بود.میانگین سن شروع مصرف سیگار 6 ± 6/21 سال بود .میزان کاهش وابستگی به نیکوتین در گروه مداخله25/5 و درگروه شاهد 86/4 می باشد. در مورد میزان ترک سیگار پس از سه ماه 6/79% از ا فراد گروه مداخله و 66% از افراد گروه شاهد سیگار را ترک کرده بودند که درصد ترک گروه مداخله بیشتر بود اما اختلاف آنها از نظر آماری معنی دار نبود.   نتیجه گیری : نتایج این مطالعه بیانگر آن است که برای ترک سیگار در این گروه از بیماران لازم است برنامه های آموزشی همراه با مشاهده تصویر اجرا گردد.

Title: کاربرد پودر معمولی گل گیاه تلخه به عنوان جاذب بیولوژیکی طبیعی در حذف کروم شش ظرفیتی از فاضلاب سنتتیک

http://tbj.ssu.ac.ir/article-1-179-fa.html

سابقه و اهداف: یکی از مهمترین نگرانیهای اخیر در مورد فاضلابهای صنعتی ورود فلزات سنگین به منابع آب و محیط زیست می باشد. کروم به صورت اکسیدهای سه و شش ظرفیتی در فاضلاب صنایعی نظیر آبکاری و دباغی یافت می شود. کروم شش ظرفیتی دارای مخاطرات بهداشتی اثبات شده ای مانند سرطانزایی می باشد. هدف از این مطالعه بررسی کاربرد پودر معمولی گل گیاه تلخه در حذف کروم شش ظرفیتی از فاضلاب سنتتیک می باشد.   روش بررسی: در این تحقیق اثر غلظت اولیه کروم، دز جاذب، زمان واکنش و pH بر حذف کروم شش ظرفیتی بررسی شد. جاذب مورد نظر در شرایط آزمایشگاهی تهیه و با استفاده از الکهای استاندارد ASTM با اندازه های مش بین 60-40 دانه بندی شد. مطالعه بر روی نمونه سنتتیک حاوی غلظت های 2و10 میلی گرم در لیتر کروم انجام گرفت. غلظت مقادیر مجهول کروم شش ظرفیتی به روش اسپکتروفتومتری در طول موج 540 نانومتر بر اساس روش ارائه شده درکتاب روشهای استاندارد آزمایشهای آب و فاضلاب تعیین گردید.   یافته ها: با توجه به نتایج با افزایش دز جاذب و زمان واکنش، مقدار حذف آلاینده افزایش و با افزایش pH محلول و غلظت اولیه کروم مقدار حذف آلاینده کاهش می یابد. داده های این تحقیق نشان داد حد اکثر میزان ظرفیت جذب پودر معمولی گل گیاه تلخه، برای غلظتهای اولیه کروم 2 و 10 میلی گرم در لیتر به ترتیب 66/1 و72/5 میلی گرم بر گرم بوده است.   نتیجه گیری: پودر معمولی گل گیاه تلخه جاذبی مؤثر در حذف کروم از فاضلاب سنتتیک می باشد. هر چند این گیاه به عنوان علف هرز شناخته شده اما ساختار بسیار متخلخل گل این گیاه توانایی جذب خوبی را ایجاد نموده است. به علاوه کاربرد این جاذب از نظر آماده سازی و هزینه بسیار ساده و ارزان بوده و کاربرد آن در مقایسه با بسیاری دیگر از جاذبهای طبیعی و مصنوعی دارای اولویت است.

Title: حذف گاز متیل یدید از هوا در بسترهای کربن فعال اصلاح شده با نمک های آمینی

http://tbj.ssu.ac.ir/article-1-180-fa.html

سابقه و اهداف: در میان گازهای هسته ای خروجی از نیروگاه های هسته ای ترکیبات ید رادیواکتیو از اهمیت بالایی پیش از تخلیه به محیط زیست برخوردار هستند. این مطالعه با هدف ساخت بسترهای جذبی کربن فعال اصلاح شده با نسبت های مختلف از نمک های آمینی و ارزیابی ظرفیت و کارایی جذب آنها جهت جذب بخارات متیل یدید انجام شد.   روش بررسی : کربن فعال اصلاح شده با نمک های آمینی، TEDA و HMTA در نسبت های 5/2، 5، 5/7 ، 10و بیشتر از ده درصد وزنی ماده اصلاح کننده به ازای هر گرم جاذب به روش محلولی ساخته شدند.مطالعات جذب سطحی در شرایط دمای محیط و رطوبت نسبی صفر و 65 درصد در غلظت 19-10 پی پی ام متیل ید با بهره گیری از پایلوت جذب سطحی انجام شد.   یافته ها : ظرفیت جذب متیل یدید بر روی کربن فعال در رطوبت نسبی 65% درحدود 92% کاهش یافت، از اینرو جذب اندک متیل یدید بر روی کربن فعال فقط در شرایط با رطوبت نسبی پایین امکان پذیر بوده و نمی تواند به عنوان جاذب قابل اعتمادی در تصفیه جریان های هوای آلوده به متیل یدید در رطوبت نسبی بالا مطرح باشد.ظرفیت جذب متیل یدید بر روی کربن فعال اصلاح شده با HMTA در شرایط با رطوبت نسبی بالا افت قابل توجهی داشته و به حدود 7/1 میلی گرم متیل یدید به ازای هر گرم جاذب می رسد که تفاوت چندانی با ظرفیت جذبی کربن فعال معمولی ندارد.   نتیجه گیری : به نظر می رسد کربن فعال اصلاح شده با TEDA در نسبت اصلاح سازی 8-4 درصد از ظرفیت جذبی مناسبی برای متیل یدید برخوردار بوده و می تواند به عنوان جاذب قابل اعتمادی در شرایط جوی مختلف استفاده شود.

Title: تولید ورمی کمپوست در مقیاس خانگی با کرم خاکی

http://tbj.ssu.ac.ir/article-1-181-fa.html

سابقه و اهداف: امروزه فرایند تولید ورمی کمپوست بعنوان یکی از روشهای بسیار مناسب از لحاظ اقتصادی، بهداشتی و سازگاری با محیط زیست برای تثبیت مواد زاید آلی مطرح است. هدف از این مطالعه، تولید ورمی کمپوست از زباله شهری توسط کرم خاکی( (Eisenia fetida در مقیاس خانگی می باشد.   روش بررسی: در این مطالعه که بصورت تجربی انجام شد، در ابتدا مخزن پلاستیکی تولید ورمی کمپوست تهیه شد و سپس با استفاده از بستر اولیه، خاک نرم، مواد زائد قابل کمپوست و کرمهای ایزنیا فوئتیدا اقدام به بارگذاری آن گردید و بعد از 30 و 90 روز، اقدام به نمونه برداری شد. در نمونه های مذکور پارامترهای درصد مواد آلی، درصد خاکستر، pH ، کل کربن، ازت کل، درصد فسفر، هدایت الکتریکی و رطوبت اندازه گیر شد.   یافته ها: نتایج نشان داد که درصد مواد آلی، درصد خاکستر، درصد کربن کل، درصد ازت کل، نسبت کربن به ازت، درصد فسفر، هدایت الکتریکی، درصد رطوبت و pH کمپوست رسیده(90 روزه) به ترتیب برابر با 8/2 ± 42%، 17/0 ± 53%، 17/0 ± 22%، 003/0 ± 12/1%، 25/0 ± 20، 26/0 ± 8/2%، m c/ S µ 200 ± 12000، 5/5 ± 56% و 2/0 ± 3/8 حاصل شد که تمام پارامترهای مورد سنجش، مطابق با استاندارد بود.   نتیجه گیری: با توجه به نتایج می توان نتیجه گرفت که صاحبان منازل با استفاده از امکانات ساده و محلی، می توانند ورمی کمپوست با کیفیت بالا و مطابق با استاندارد تولید نمایند که می تواند از نظر زیست محیطی و اقتصادی کمک شایانی به سیستم مدیریت جامع مواد زاید جامد شهری نموده و سبب کاهش بیش از 70-60% پسماند خانگی در مبدأ گردد.

Title: بررسی عوامل موثربر غلظت نیترات منابع آب شرب زیرزمینی شیراز با استفاده از سیستم اطلاعات جغرافیایی(GIS)

http://tbj.ssu.ac.ir/article-1-182-fa.html

سابقه و اهداف : نیترات یکی از شایع ترین آلوده کننده های آبهای زیرزمینی است . در صورت مصرف آب آلوده به نیترات توسط نوزادان، سبب کاهش اکسیژن در بدن آنان شده و نهایتا باعث مرگ آن ها می گردد. هدف از انجام این مطالعه اندازه گیری میزان نیترات و نیتریت ، علل ومنشا آن و پهنه بندی غلظت نیترات در منابع آب شرب زیرزمینی شیراز با استفاده از نرم افزار 9/3 Arcview GIS می باشد .   روش بررسی : در این تحقیق توصیفی220 نمونه آب از 55 حلقه چاه شهر شیراز در فصل پر بارش وکم بارش به صورت منظم از تمام چاهها برداشت شد. آزمایش نیترات و نیتریت، با دستگاه اسپکتوفتومتری انجام شد، نتایج بدست آمده در نرم افزار SPSS و Arcview gis9.3 پهنه بندی و تحلیل شد.   یافته ها : نتایج نشان داد غلظت نیترات در منابع آب شرب زیرزمینی شیراز در محدوده 4 تا 72 میلی گرم بر لیتر و میانگین 65/31 میلی گرم بر لیتر بوده است. 60% نمونه ها دارای غلظت نیترات بالاتر از 20 میلی گرم بر لیتر بود و در 16% نمونه ها غلظت نیترات بالاتر از حد استاندارد بدست آمد.   نتیجه گیری : بر اساس نتایج با افزایش عمق چاه غلظت نیترات کاهش می یابد . نقشه های بدست آمده نشان دادند، غلظت نیترات از غرب دشت شیراز به شرق در حال افزایش است و مهمترین منبع آلودگی چاههای شرب شهر شیراز فاضلاب شهری وخانگی می باشد. حرکت آب از آبخوان رسوبی به آبخوان آهکی از عوامل موثر بر آلودگی چاههای این منطقه می باشد.

Title: اعتبار سنجی محتوایی و ارزیابی پایداری درونی ابزار مبتنی بر تجربه سنجش مستقیم ناامنی غذایی سالمندان برای جامعه ایرانی

http://tbj.ssu.ac.ir/article-1-183-fa.html

سابقه و اهداف: امنیت غذایی یکی از مسائل مهم بهداشتی، اجتماعی، اقتصادی و سیاسی کشورهای مختلف به شمار می‌رود. در این میان برخی از ویژگی‌های خاص سالمندان آنها را بیشتر از افراد عادی در معرض ناامنی غذایی قرار می‌دهد. مطالعه حاضر با توجه به نبود ابزار معتبر برای سنجش امنیت غذایی سالمندان در ایران با هدف تعیین اعتبار محتوایی و ارزیابی پایداری درونی ابزار سنجش ناامنی غذایی این گروه طراحی و اجرا شده است.   روش بررسی: در این مطالعه پرسش‌نامه 8 آیتمی سنجش ناامنی غذایی سالمندان بر اساس دستورالعمل مقیاس اندازه‌گیری دسترسی ناامنی غذایی خانوار (ویرایش سوم) ( HAIFS ) ( Household Food Insecurity Access Scale Indicator Guide ) و راهنمای فنی ایجاد ابزار سنجش مستقیم و مبتنی بر تجربه ناامنی غذایی خانوار طی پنج مرحله مورد اعتبار سنجی قرار گرفت.   یافته‌ها: پرسش‌نامه در هر مرحله بر اساس نظرات متخصصان و افراد آگاه، تقریرات نمایندگان گروه هدف و نظرات اصلاحی محققان اصلی بر ترجمه مجدد پرسش‌ها مورد اصلاح قرار گرفت. برای پایداری درونی، آلفای کرونباخ در این بررسی در حد مطلوب 858/0 بدست آمد. در 20 نمونه مورد بررسی نیز 7 سالمند به لحاظ امنیت غذایی امن، 3 سالمند ناامن بدون گرسنگی، 5 سالمند ناامن با گرسنگی متوسط و 5 سالمند ناامن با گرسنگی شدید طبقه بندی شدند.   نتیجه‌گیری: مطالعه حاضر پیش ابزار مناسبی برای تعیین وضعیت امنیت غذایی سالمندان در جامعه ایرانی ارائه می‌دهد که به لحاظ اعتبار محتوایی و پایداری درونی از اعتبار مناسبی برخوردار است. با این وجود پیشنهاد می‌شود با در نظر گرفتن متغیرهای عمده تأثیرگذار بر وضعیت امنیت غذایی و بکارگیری این پیش ابزار در جامعه‌ای بزرگتر اعتبار سازه‌ای و ملاکی نیز برای آن تعیین شود.

Title: بررسی تاثیر آموزش سبک مقابله ای اسلامی بر افزایش شادکامی والدین کودکان استثنایی

http://tbj.ssu.ac.ir/article-1-184-fa.html

سابقه و اهداف: مطالعات نشان داده اند که والدین کودکان استثنائی از شادکامی و رضایت زندگی کمتری برخوردارند.سبک های مقابله ای یکی از مهمترین عوامل روانی اجتماعی است که رابط بین فشار و بیماریهای روانی می باشد. از طرفی رابطه معناداری بین شادکامی وباورهای دینی وجود دارد .لذا این مطالعه با هدف بررسی تاثیر آموزش سبکهای مقابله ای اسلامی بر میزان شادکامی والدین کودکان استثنائی در شهرستان خاتم (هرات و مروست) انجام شد.   روش بررسی :مطالعه حاضر یک مطالعه تجربی است. حجم نمونه 24زوج بودکه از بین والدین کودکان استثنائی شهرستان خاتم انتخاب شدند وبه طور تصادفی در دو گروه آزمون و کنترل قرار گرفتند.آموزش سبک مقابله اسلامی به صورت گروهی، برای گروه آزمون اجرا شد.پس از گذشت یک ماه داده های حاصل از پژوهش با استفاده از آمار توصیفی و استنباطی مورد تجزیه و تحلیل قرار گرفت.   یافته ها: آموزش سبکهای مقابله اسلامی، شادکامی والدین کودکان استثنائی را افزایش داد به نحوی که میانگین شادکامی از 64/4 ± 41/26 قبل از مطالعه به 19/9 ± 04/56 بعد از مطالعه و به 63/9 ± 55 بعد از پیگیری یک ماهه رسید.   نتیجه گیری : با توجه به ثمر بخش بودن استفاده از سبکهای مقابله اسلامی در افزایش شادکامی ،آموزش این سبک ها در راستای کاهش فشارهای روانی ومقابله با آنها باید صورت گیرد.

Title: ارزیابی عملکرد انواع فرآیندهای فنتونی در ترکیب با فرآیند انعقاد و لخته‌سازی متداول در تصفیه شیرابه محل دفن

http://tbj.ssu.ac.ir/article-1-185-fa.html

سابقه و اهداف: فرآیندهای اکسیداسیون پیشرفته نظیر فنتون و فتو فنتون به طور موثری برای اکسیداسیون ترکیبات آلی مقاوم و تبدیل آنها به مواد بی‌خطر در شیرابه کاربرد دارند. با این حال ، اطلاعات اندکی در مورد استفاده از فرآیند فنتون اصلاح شده در تصفیه شیرابه وجود دارد. بنابراین، هدف از این مطالعه تصفیه شیرابه زباله های شهری توسط سه فرآیند فوق در ترکیب با فرآیند انعقاد و لخته‌سازی به عنوان پیش‌تصفیه می‌باشد.   روش بررسی: این مطالعه یک مطالعه تجربی است که با استفاده از سیستم پایلوت آزمایشگاهی انجام شد. شیرابه مورد استفاده از دفن زباله شهری قائم شهر جمع آوری شد. کلیه آزمایشات و آنالیز نمونه به روش کتاب استاندارد آزمایشهای آب و فاضلاب انجام شد.   یافته‌ها: نتایج این تحقیق نشان داد که فرآیند انعقاد و لخته‌سازی متداول با کلرور فریک در شرایط بهینه حداکثر حدود45 درصد COD را حذف می‌نماید. ترکیب فرآیند پیش‌تصفیه با فرآیندهای فنتونی راندمان حذف را بطور قابل توجهی افزایش می‌دهد. بطوریکه راندمان حذف COD در شرایط یکسان در سه فرایند فنتون، فتوفنتون و فنتون اصلاح شده به ترتیب 89، 45/85 و 82/81 درصد بود. در هرسه فرایند میزان تجزیه‌پذیری شیرابه ( BOD5/COD ) افزایش می‌یابد که در فرآیند فتوفنتون بیش از دو فرایند دیگر می‌باشد. نتیجه‌گیری: فرآیند فتو-فنتون با پیش‏تصفیه دارای بیشترین راندمان در تصفیه شیرابه می‏باشد. اما هزینه و مشکلات بهره‌برداری آن بیش از دو فرآیند دیگر می‌باشد. کارایی فنتون اصلاح شده کمتر از دو فرایند دیگر است اما این فرایند در pH بالاتری عمل می‌کند و مشکلات تنظیم pH آن کمتر می‌باشد.

Title: بررسی و مقایسه عملکرد سولفات آلومینیوم و پلی آلومینیم کلراید (PAC) درتصفیه فاضلاب صنعت روغن‌کشی زیتون

http://tbj.ssu.ac.ir/article-1-186-fa.html

سابقه و اهداف: استخراج روغن زیتون در جهان یک فعالیت مهم اقتصادی محسوب می شود. اثرات زیست محیطی فاضلاب صنعت روغن زیتون مربوط به محتویات شیمیایی و مواد آلی بوده که مقاوم به تجزیه بیولوژیکی هستند. فاضلاب زیتون به علت وجود ترکیبات بازدارنده فنولی، سمیت برای میکروارگانیسم ها در واحدهای تصفیه فاضلاب شهری، بوی نامطبوع ، COD بالا، BOD بالا، جامدات معلق بالا، ترکیبات مقاوم به تجزیه بیولوژیکی، پتانسیل تهدیدی برای منابع آب سطحی و زیرزمینی محسوب می گردد. تاکنون مطالعات متعددی در زمینه تصفیه فاضلاب صنعت روغن زیتون انجام شده است. هدف از این مطالعه نیز تعیین کارآیی فرآیند انعقاد جهت کاهش بار آلودگی و بهبود قابلیت تجزیه پذیری بیولوژیکی فاضلاب روغن زیتون می باشد.   روش بررسی: این مطالعه در مقیاس آزمایشگاهی و به صورت ناپیوسته بر روی فاضلاب واقعی انجام شد. فرآیند انعقاد با استفاده از مواد منعقد کننده آلوم و پلی آلومینیوم کلراید انجام گرفت و میزان حذف پارامترهای COD ، کل ترکیبات فنلیک و TSS مورد بررسی قرار گرفت.   یافته‌ها: نتایج نشان داد که بالاترین راندمان حذف آلاینده ها در pH و دوز بهینه پلی آلومینیوم کلراید به ترتیب 7 و mg/L 1000 حاصل گردید و در این شرایط میزان حذف COD ، کل ترکیبات فنلیک، TSS و کدورت توسط پلی آلومینیوم کلراید به ترتیب 3/88، 2/90 و 2/99 درصد حاصل گردید. همچنین اندازه گیری نسبت BOD/COD نشان داد که این مقدار بعد از فرآیند از 14/0 به 58/0 افزایش یافت. نتیجه‌گیری: این مطالعه نشان داد که فرآیند انعقاد با استفاده از ماده منعقد کننده پلی آلومینیوم کلراید می‌تواند به عنوان روش پیش تصفیه موثر و اقتصادی در کاهش بار آلایندگی این نوع فاضلاب به کار گرفته شود.

Title: پاکسازی زیستی آب های آلوده به نفت خام توسط باسیلوس های جدا شده ازحوضچه های نفتی

http://tbj.ssu.ac.ir/article-1-187-fa.html

سابقه و اهداف: آب های آلوده به نفت خام یا ترکیبات نفتی یکی از معضلات محیط زیست اند. پاکسازی زیستی یک روش ساده و اقتصادی برای تصفیه آب های آلوده به این آلاینده هاست. هدف از این تحقیق بررسی قدرت تجزیه نفت خام توسط باسیلوس های جداسازی شده از آب های آلوده به نفت خام پالایشگاه نفت اصفهان می باشد.   روش بررسی: برای این منظور از آب های آلوده به نفت خام در پالایشگاه نفت اصفهان استفاده شد. نمونه های برداشت شده بر روی محیط MSM کشت داده شده و به آن 10000 ppm نفت به عنوان تنها منبع کربنی باکتری ها اضافه شد.این محیط کشت بر روی تکان دهنده ای با دور 150 rpm و دمای 35 درجه سانتی گراد گرماگذاری شد. برای جداسازی باکتری‌های مولد بیوسورفکتانت، از بررسی فعالیت همولیتیک به عنوان اولین معیار جداسازی و غربالگری استفاده شد. برای بررسی قدرت تجزیه نفت خام هر3 سویه خالص شده و کنسرسیوم این3 سویه تست های IR (مادون قرمز) و )GC-MS گاز کروماتوگرافی-اسپکترومتر) گذاشته شد. باسیلوس های خالص شده با روش های تشخیصی بیوشیمیایی و ملکولی شناسایی شدند.   یافته ها: از بین 12 سویه جداسازی شده، 8 سویه قادر به لیز نمودن اریتروسیت‌های گوسفند و تولید هاله همولیز برروی محیط بلاد آگار بودند. سویه‌های دارای فعالیت همولیتیک برای مطالعات بعدی انتخاب گردیدند. کشش سطحی 3 سویه قابل پذیرش بود.کنسرسیوم این 3 سویه نیز از نظر تولید بیوسورفکتانت بررسی شد ومقادیر 38 ،30،34 mN/m برای 3 سویه خالص شده و 28 mN/m برای کنسرسیوم این 3 سویه بدست آمد. هنگامی‌که توا لی فوق در برنامه BLAST قرار گرفت، نتایج نشان دهنده تشابه 8/99 درصد باکتری های جدا شده با Bacillus subtilis و Bacillus cereus بودند.دو سویه Bacillus subtilis شناسایی شده تنها در 6 نوکلئوتید با هم تفاوت داشتند.   نتیجه گیری: بررسی های انجام شده نشان داد، عملکرد کنسرسیوم در تجزیه زیستی بسیار مؤثرتر از کشت باسیلوس ها به تنهایی بوده، در واقع کنسرسیوم باسیلوس ها با قدرت بیشتری نسبت به باسیلوس ها به تنهایی در تجزیه زیستی نفت خام در آب های آلوده عمل کرده است.

Title: بررسی اضطراب دندانپزشکی دربیماران مراجعه کننده به مطب های دندانپزشکی بخش خصوصی شهرستان مشهد

http://tbj.ssu.ac.ir/article-1-188-fa.html

سابقه واهداف: اضطراب بیماران و عدم مراجعه آنان برای برخورداری از خدمات دندانپزشکی ممکن است تأثیرنامناسبی بر شاخصهای توسعه جامعه داشته باشد. مطالعات انجام شده در این زمینه محدود می باشند و نتایج مطالعات در این زمینه دارای تناقضات متعدد است، لذا این مطالعه به منظور تعیین وضعیت اضطراب دندانپزشکی در بیماران مراجعه کننده به مطب های بخش خصوصی دندانپزشکی شهرستان مشهد در سال 1390انجام شد.   روش بررسی: در این مطالعه توصیفی، بطور تصادفی تعداد 400 نفر ازمراجعه کنندگان به بیست مطب دندانپزشکی شهر مشهد که آنها نیز بطور تصادفی انتخاب شده بودند، در مطالعه وارد شدند. اطلاعات با استفاده از مقیاس اضطراب دندانپزشکی Dental Anxiety Scale) ) جمع‌آوری گردید. پایایی و روایی پرسشنامه درمطالعات قبلی مورد سنجش قرار گرفته و تایید گردیده است. داده‌ها با استفاده از نرم‌افزار SPSS و به‌کارگیری آزمونهای آماری تی، ANOVA ، ضریب همبستگی پیرسون و اسپیرمن مورد تجزیه و تحلیل قرار گرفت.   یافته‌ها: در این مطالعه 8/16% از افراد دارای اضطراب خفیف، 5/58% دارای اضطراب متوسط و 8/24% دارای اضطراب شدید دندانـپزشکی بودند. میانگین نمـره اضطـراب در زنـان (91/3 ± 14/12 ( بیـشتر از مـردان (89/3 ± 19/9 ( بود (000/0 p= ). به لحاظ وضعیت تأهل بیشترین میانگین نمره اضطراب در افرادمطلقه و همسر مرده (4 ± 39/13) مشاهده شد (000/0 p= ). از لحاظ شغلی بالاترین میانگین نمره اضطراب در افراد خانه دار (77/3 ± 89/12) دیده شد. بین نمره اضطراب دندانپزشکی و سن (013/0 p= ، 125/0- r= )، دفعات مراجعه به مطب دندانپزشکی ( 004/0 p= ، 143/0- r= ) سطح تحصیلات ( 001/0 p< ، 319/0- ρ= ) ونیز سطوح مختلف درآمد ( 001/0 p< ، 415/0- ρ= ) همبستگی معکوس ومعنی داری وجودداشت. نتیجه‌گیری: از آنجا که در مطالعه حاضر میزان اضطراب دندانپزشکی در زنان و بخصوص زنان خانه دار بیشتر دیده شد، ونیز با سن وسطح سواد و سطح درآمد همبستگی معکوس داشت، افراد با ویژگیهای فوق بایستی دربرنامه های آموزشی و مداخله ای در این زمینه، موردتوجه بیشتری قراربگیرند
